# Digital health: A sociomaterial approach

**DOI:** 10.1111/1467-9566.13538

**Published:** 2022-08-28

**Authors:** Benjamin Marent, Flis Henwood

**Affiliations:** ^1^ University of Sussex Business School University of Sussex Brighton UK; ^2^ School of Humanities and Social Science University of Brighton Brighton UK

**Keywords:** agential realism, digital health, digital sociology, digital transformation, governance, relationality, responsible innovation, sociomateriality

## Abstract

The notion of *digital health* often remains an empty signifier, employed strategically for a vast array of demands to attract investments and legitimise reforms. Rather scarce are attempts to develop digital health towards an analytic notion that provides avenues for understanding the ongoing transformations in health care. This article develops a sociomaterial approach to understanding digital health, showing how digitalisation affords practices of health and medicine to cope with and utilise the combined and interrelated challenges of increases in quantification (data‐intensive medicine), varieties of connectivity (telemedicine), and unprecedented modes of instantaneous calculation (algorithmic medicine). This enables an engagement with questions about what forms of knowledge, relationships and control are produced through different manifestations of digital health. The paper then sets out, in detail, three innovative strategies that can guide explorations and negotiations into the type of care we want to achieve through digital transformation. These strategies embed Karen Barad’s concept of *agential cuts* suggesting that responsible cuts towards the materialisation of digital health require participatory efforts that recognise the affordances and the generativity of technology developments. Through the sociomaterial approach presented in this article, we aim to lay the foundations to reorient and sensitise innovation and care processes in order to create new possibilities and value‐centric approaches for promoting health in digital societies as opposed to promoting digital health per se.

## INTRODUCTION

The notion of *digital health* has become widely used in reference to emerging technologies adopted across various practices of health and medicine. As with all new technologies, the promotion and uptake of digital health technologies is often underpinned by promissory discourses. These mobilise utilitarian and democratic arguments to praise the potentials of digital transformation for increasing efficiency of health services and for empowering citizens to take‐up new responsibilities in their care. Pickersgill (2019) analyses digital health as *performative nominalism* that supports professional projects in the constitution of novel fields and the consolidation of authority. The notion of digital health often remains an empty signifier, employed strategically for a vast array of demands to attract investments and legitimise reforms. Rather scarce are attempts to develop digital health towards an analytic notion that provides avenues for understanding the ongoing transformations in health care.

Marent and Henwood (2021) conceptualised digital health as an umbrella term that encompasses four broad fields of practice that have emerged since the 1980s, accompanied by sociological research. Sociologists have investigated how *telemedicine* enabled care at distance through the utilisation of information and communication technology (Finch et al., 2008). The new opportunities of searching and exchanging health information through the World Wide Web were intensively studied under the notion of *eHealth* (Kivits, 2013). More recently, policy makers are advocating *mHealth*. This involves practices of using mobile digital devices for health‐related reasons, which became an important focus for sociological critique (Lupton, 2012). *Algorithmic medicine* is the latest disruption, whereby advances in data science and artificial intelligence are incorporated in highly complex processes of prediction and medical diagnosis (Petersen, 2019).

By engaging with digital health as important and manifold phenomena, the sociology of health and illness has counteracted the promises of numerous industrial ventures and policy claims in order to reveal the social contours of technological change. Yet, sociological research has not been utilised solely to understand the consequences of digital transformation in health. As outlined in our Special Issue for this journal, *Digital Health*: *Sociological Perspectives* (Henwood & Marent, 2019), processes of digitalisation also challenge core concepts of sociological theory. Therefore, contributions in the Special Issue developed further important notions such as trust (Petersen et al., 2019), reflexivity (Numerato et al., 2019), intimacy (Piras & Miele, 2019), or accountability (Schwennesen, 2019). Practical and theoretical challenges of digitalisation addressed across contributions of the Special Issue reflected, according to our synthesis (Henwood & Marent, 2019), reconfigurations of knowledge, connectivity, and control. In this article, we want to continue this theoretical endeavour to elaborate an understanding of digital health that embeds these key reconfigurations of practices. This understanding can enable sociologists to re‐signify prior research and provide promising novel lines of inquiry.

For this endeavour we elaborate the sociomaterial approach proposed in the Special Issue, building on a long tradition in science and technology studies focussing on the contingent intertwining of the social and the material (Latour, 2005; Pickering, 1995; Suchman, 2007). This radical relational ontology has been well captured by Karen Barad’s agential realism (Barad, 2007) and we build on her work here. Barad’s relational ontology, as Lemke (2021) points out, departs from other strands of new materialist theorising that propose an object‐oriented ontology assuming the pre‐existence of material artefacts (cf. Harman, 2016) or advance vitalist ideas that identify agency as quality inherent in material beings (cf. Bennett, 2010). While each different new materialist direction can offer important insights to the understanding of digital health—for example, demonstrated by Lupton (2019) developing Bennett’s vital materialism—we focus on demonstrating the value and applicability of Barad’s agential realism and its recent advances in sociology, science and technology studies and information systems research (Cecez‐Kecmanovic et al., 2014; Lemke, 2021; Schultze et al., 2020).

Taking a sociomaterial approach to digital health involves refusing a fixation on matter as a property innate in technologies and, instead, exploring how technologies *come to matter* and generate possibilities through their enactment in practices. A focus on sociomaterial practices lays the foundations for understanding how functionalities and meanings of technologies shift continuously across spatiotemporal situations. Digital health and its situated meanings and possibilities materialise through practices. As we go on to argue, digital health practices have to cope with and utilise the combined and interrelated challenges of increases in quantification, varieties in connectivity and unprecedented modes of instantaneous calculation. Respective reconfigurations in the creation of knowledge, the formation of relationships and the conduct of control provide important foci for understanding digital health. Moreover, we outline how the onto‐epistemological positioning of the sociomaterial approach is deeply entangled with ethical concerns and questions of how to engage in interventionist research. Thereby, we discuss three strategies to direct the conduct of digital health innovation projects.

The paper proceeds as follows. First, sociomateriality is outlined as an ontological position distinct to positivistic and interpretivist approaches applied in the study of technology, including digital health. Second, digital health is conceptualised as sociomaterial practices that process manifold forms of quantification, connectivity, and instantaneity and involve reconfigurations of knowledge, relationships and control that are important analytical foci to understand digitalisation processes. Third, key concepts from sociomaterialism such as ontological politics, relationality, and performativity are brought into dialogue with concepts of participation, affordances, and generativity to generate innovative strategies for conducting research *within* digital health innovation projects. We conclude by arguing that the foci and strategies set out by the sociomaterial approach can lay the foundations to reorient and sensitise innovation processes in order to create new possibilities and value‐centric approaches for promoting health in digital societies as opposed to promoting digital health per se.

## ONTOLOGICAL TURNING: FROM FIXED TECHNOLOGIES TO PERFORMATIVE PRACTICES

What *is* digital health? This question now comes across frequently in our everyday lives as health sociologists. It is most commonly framed by a substantialist ontology, where the questioner often seeks help for making sense of the number and plurality of digital technologies that are being embedded in health practices. From this substantialist stance, digital technologies are approached as objects with fixed boundaries and essential properties. It is assumed that digital technologies have an existence independent of humans, but notwithstanding impacting or mediating human affairs.

Schultze et al. (2020) have recently elaborated how this substantialist stance has dominated positivistic and interpretivist approaches applied in the study of technology. The positivistic approach separates the subject and object and further classifies both according to their essential and distinguishing properties. Of interest is the specific causal relationship between an object with inherent features and a subject with innate demographics and competences. This relationship is most often configurated in terms of technological determinism, where society is seen as a product of technological advances. The positivistic lens aims to magnify the physical and social reality through the production of homologous copies of the original entities and linear causalities.

Interpretivist stances, by contrast, do not approach technological objects as simply given. Rather, technologies are recognised as being constructed in and through human actions. The interpretivist’s interest is to understand how human subjects make sense of technological objects and how human‐technology interactions enable or restrict social activities. From this perspective, a subject and an object are still conceived exterior to their relation. However, it is acknowledged that empirical and theoretical accounts of such entities and their relations are situated and partial representations. In this way, the *mirror*—rather than the positivist’s *lens*—portrays the interpretivist’s apparatus (Østerlund et al., 2020). Operating through the mirror, interpretivist researchers have to account for their subjective positioning and potential distortions that are leading to the reflection of the object of their study.

More recently, many technology studies and information systems researchers have been reconfiguring their ontological foundation in a turn towards *sociomateriality* (Cecez‐Kecmanovic et al., 2014; Schultze et al., 2020). For example, Barad’s (2007) agential realism has been adopted as a novel framework to explain the entanglement of phenomena such as digital health. Applied to digital health, this form of realism would not be concerned with accurately representing a reality of independent objects (e.g. health apps) and human subjects (e.g. patients/users), but instead would recognise the entanglement of non‐human and human elements in the constitution of phenomena such as, for example, online consultations through an app. From this ontological stance, the identities and properties of a certain health app and its patient user are ‘not taken as given and preexisting before entering into relations’ (Cecez‐Kecmanovic et al., 2014, p. 566). Rather, these entities and their properties and meanings materialise through dynamic *intra‐actions*. Through this neologism, Barad (2007) aims to capture the process through which things, such as an app or a patient, become constituted in mutual emergence. These things—for Barad (ibid.)—are not *things‐in‐themselves* (connected through interactions) but *things‐in‐phenomena* that materialise through the agentic processes of intra‐action. The ontological shift from *things* or *technologies* to *phenomena* recognises the ‘inseparability of observer and observed’ (Barad, 2007, p. 139). This requires attention to the performativity of practice that is reconfiguring the possibilities for human and non‐human agencies to act and become enacted in certain ways. ‘(T)he contours of social and material agency are mangled in practice’ (Pickering, 1995, p. 23). Their relationship is one of ‘mutual entailment. Neither is articulated/articulable in the absence of the other’ (Barad, 2003, p. 822). The sociomaterial turn thus acknowledges that ‘there is no social that is not also material, and no material that is not also social’ (Orlikowski, 2007, p. 1437).

In this article, we develop Barad’s agential realist approach, to generate an understanding of how the digital plays a role in reconfiguring *matters of care* (Puig de La Bellacasa, 2017). It is acknowledged that interpretations and uses of digital technologies as well as claims about their capacity and effects are inescapably contingent. Thus, digital health is conceptualised as dynamic phenomenon that materialises through ‘open‐ended practices’ (Barad, 2007, p. 170). This agential realist account provides not a definite answer to what digital health *is*, but, as we will elaborate in the following, opens new questions and foci to approach and engage in the ongoing reconfigurations of digital health practices.

## APPROACHING DIGITAL HEATH PRACTICES

How can we *approach* digital health? If Barad’s (2007) agential realism shifts the focus away from technological features as well as their interpretations by human actors, how can we advance our interest in digital health and orchestrate its materialisations as researchers engaged in innovation projects? Barad (2007) offers a posthumanist, performative account by which we can approach digital health as contingent and as a practical accomplishment. Neither humans nor material artefacts are assumed to be the sole locus of agency or the authors of meaning. Barad’s posthumanist performative account refuses the fixation of matter as a property innate in an object or meaning as something achieved through the intentionality of a subject. Instead, she emphasises that ‘(m)eaning is made possible through specific material practices’ (Barad, 2007, p. 148).

Practices handle complexity and bring phenomena into being by *boundary‐making*. As Barad (2007, p. 139) emphasises: ‘It is through specific agential intra‐actions that the boundaries and properties of the components of phenomena become determinate and that particular concepts (i.e. particular material articulations of the world) become meaningful.’ Boundaries are drawn through *agential cuts*, which select certain possibilities from a horizon of potentialities. Thus, phenomena such as digital health come to ‘matter’—in both senses of the word—through agential intra‐actions where certain possibilities are actualised and others are excluded. For example, such processes of the materialisation of meaning have been empirically investigated through the case of regulatory agencies, which play an important role in classifying and fixing the boundaries of emerging technologies. Alex Faulkner has outlined how regulatory practices enact and determine the ‘medical deviceness’ of technologies and thus transform them into the subject of governance (Faulkner, 2008). More recently, Lievevrouw and colleagues have charted the standard‐making process of the US Food and Drug Administration’s (FDA) digital health policy and outlined its challenges and tensions to elaborate the ‘world’s first attempt to demarcate the boundary between lifestyle and medical digital health technologies’ (Lievevrouw et al., 2021).

Regulatory agencies are a vivid example of how a given definition of digital health constitutes boundaries and thereby permit or prohibit novel products from market entry. It is important to recognise that inclusions as well as exclusions constitute new spaces of agency and questions of accountability (Barad, 2007). In this way, the tech industry and business representatives often engage in attempts to challenge regulatory frameworks and lobby for more innovation‐friendly policy. Facing such demands and struggles to define digital health, the FDA shifted its focus from the individual product or the platform on which it resides to the product’s intended use (risk‐based assessment) as well as to the applying company (organisational culture). Through this new regulatory framework, the FDA pulled back as gatekeeper for many health applications (if these become classified ‘low risk’) and offered simplified approval procedures for applications classified as ‘lower risk’. Furthermore, the FDA started a pre‐certification pilot to assess the *culture of quality* of the applying companies instead of going through each individual product’s risk assessment. Lievevrouw et al. (2021) stress that this innovation‐friendly and rapid pre‐market approval system does require a novel and extensive post‐market monitoring framework in order to ensure patient safety and quality.

In theoretical terms, sociomaterial practices produce certain uses of objects and positionings of user subjects through actualisation of certain possibilities, while there is always a horizon of undefined disposability. Therefore, in the context of digital health, the meaning of objects (e.g. by ascribing classifications or functionalities) and subject positions (e.g. by ascribing new responsibilities to patient users) can continually mutate. Barad emphasises that sociomaterial practices are ‘open ended’ and continuously reconfiguring what is possible and what makes sense to do (Barad, 2007, p. 146). The example of the FDA’s shifting regulatory practice alongside complex negotiation about what constitutes a digital health device demonstrates how digital health can be understood as phenomenon that unfolds within dynamic practices of the production of meaning in its inextricability from matter. General dimensions through which reality is enacted as meaningful are temporality, spatiality, and matter (Barad, 2007, p. 146). In the following, we outline how these dimensions of meaning are associated with central aspects of digitalisation: instantaneity, connectivity, and quantification.

Each of the following three sections start by engaging with a technological artefact that plays a significant role in the process of digitalisation: the bitstring, distributed hypertext, and silicon‐based microchips. Starting from these specific objects, we subsequently outline how the sociomaterial thinking allows to expand the understanding of digitalisation from its technological foundation to complex processes of quantification, connectivity, and instantaneity. The sections re‐signify past sociological research and point to future directions for digital health research and innovation. Through these theoretical advancements, we define digital health as sociomaterial practices of health and medicine that cope with and utilise the combined and interrelated challenges of increases in quantification (e.g. data‐intensive medicine), ubiquitous connectivity (e.g. remote access to care providers) and unprecedented modes of instantaneous calculation (e.g. algorithms applied in medical decision‐making). We show how this then suggests three foci for digital health research and innovation that can account for the dynamic reconfiguration of health knowledge (through quantification), therapeutic relationships (through connectivity) and new modes of control (through instantaneous calculation).

### Quantification: Reconfigurations of knowledge

How are digital materialities entangled in practices of quantification and reconfigurations of knowledge? As a starting point for approaching this question we could pay a visit to the computer history museum in Mountain View, California. There we would find the opportunity to glance at the simple materiality of punch cards. These pieces of stiff cardboard with holes were used throughout most of the 20st century to process digital data (Armstrong, 2019). The cards’ holes are manifestations of the intangible operations of modern computing, which is based on electronic circuits that can be on an ‘on’ or an ‘off’ mode. These modes represent the binary numbering system of bitstrings, consisting of ‘1s’ (‘on’) and ‘0s’ (‘off’). Bitstrings are the basis for all digital operations. They can be classified into programme files and data files (Faulkner & Runde, 2019). Programme files process logical operations and carry out instructions whereas data files encode and store the data used by computer programmes. Any phenomena processed by digital technologies are put into the binary logic of the bitstring and converted into quantified data. Quantification thus refers to the processes of translating analogue materiality into digital syntax.

Quantification is not a new phenomenon but rapidly expanding in the so‐called *data revolution* and recent attempts towards data‐intensive medicine (Kitchin, 2014). Data‐intensive medicine involves practices of creating, collecting, curating, and storing data, making them available for multiple potential purposes (Hoyer, forthcoming). In these practices, digital data materialise in multiple forms and representations. For example, a patient’s complex health condition becomes transformed into comparatively leaner digital data points that offer mobility and versatility in the potential forms of usage. Approaching quantification as sociomaterial practice acknowledges the entanglement of materiality in the knowing: ‘what you know is intractably connected with how you know it’ (Monteiro & Parmiggiani, 2019, p. 180). This highlights that data are not neutral entities given in the world and waiting to be gathered. Rather, data are contingent matters, brought into being through situated practices.

A vivid example of quantification and the reconfiguration of knowledge in digital health is provided by van de Wiel (2019). In the field of reproductive medicine, she has witnessed the emergence of a new form of ‘“in silico vision”, an algorithmically assisted way of seeing that makes the embryo legible, and its viability calculable’ (van de Wiel, 2019, p. 195). Van de Wiel traces the *genealogy* of data‐driven embryo selection in the contemporary global fertility sector, where the manual observation of cellular behaviours that occurred in the petri dish is increasingly replaced by automatic time‐lapse embryo imaging. Automated tracking algorithms are recording multiple visual aspects of cell development and ascribe a unique value to each embryo. In turn, the time‐lapse system matches the visual data against big data sets of historic embryo cohorts to predict the embryo’s viability. Endowed with the capabilities of producing and matching big data sets and applying model‐based reasoning, the time‐lapse imaging apparatus is able to detect cellular activities beyond what the embryologist’s medical gaze can observe through the microscope. Therefore, the ‘in silico vision’ is increasingly becoming an alternative mode of *authorised seeing*. This reconfiguration of knowledge, as van de Wiel points out, may not just result in more in vitro fertilisation success but also affect the conceptualisation and commercialisation of the assisted reproduction process and the coming into being of prenatal life.

The example illustrates how quantifying apparatuses in medicine produce phenomena. Knowing, as Lemke (2021, p. 68) states, ‘is not a passive observing practice but a material engagement with the real that takes into account what matters and what does not matter.’ In this way, data sets and algorithmic systems are performative and construct the very characteristics and descriptions of the phenomena they are purported to measure (Law, 2009). Following Barad, quantified knowing in digital health reconfigures the visual recognition of health (e.g. embryonic development) through agential cuts involved in data production (e.g. selecting embryos’ cellular temporal markers), data structuring (e.g. choices about databases, classification systems, and metadata), data distribution (e.g. granting interoperability and access across databases), and data visualisation (e.g. through scores, colours or superimposed words such as ‘high’ to ‘low’). Accounting for these sociomaterial practices highlights the ways in which data and new realities are produced. This can help challenge conceptions of health data as raw, neutral, and objective. Sociomateriality thus provides an upstream approach to ‘consider how what are taken to be data come into being’ (Jones, 2019, p. 13).

What are the implications of this for digital health research and innovation? Under the focus *reconfigurations of knowledge*, digital health research and innovation require attention to the politics of generating quantified health data and the ways in which these data are interpreted and utilised within health practices. Explorations in these directions can lead towards an understanding of how quantification produces new subject positions and reconfigures concepts of health, the body, medical authority, and expertise. For example, *digital health citizenship* produces a new set of rights and responsibility for subjects by demanding involvement in data input/production, sharing experiential knowledge or providing service feedback (Petrakaki et al., 2021). Petrakaki et al. (2021) point out that digital health citizenship moves beyond obligations for self‐care towards altruistic orientations towards the care for others by donating data to research, engaging in peer‐support and report outcome data that can inform clinical decisions or the improvement of services.

### Connectivity: Reconfigurations of relationships

The second of our three suggested foci for sociomaterial research in digital health, involves asking ‘How are digital materialities entangled in practices of connectivity and reconfigurations of relationships?’ Approaching this question requires attention to the interconnectedness of computers and people. In the computer museum we can trace the development of network computers alongside the invention of the Internet. First steps in these directions were accomplished in the in the 1960s, when the US Department of Defence funded the creation of the Advanced Research Projects Agency Network, or ARPANET. The attempt was to connect isolated computers in order to share data. This remained a difficult task before Tim Berners‐Lee developed the *distributed hypertext* in the late 1980s. Distributed hypertext provided a shared technological language for network connections among computer systems and lay the foundations for the World Wide Web in 1991. This technical language comprises three important coding formats. HyperText Markup Language (HTML) that enables shared formatting of websites, Uniform Resource Locator (URL) that allows to identify resources on the web, and standardised Hypertext Transfer Protocols (HTTP) by which information can be retrieved (Plesner & Husted, 2020). In its beginning, the Web served primarily as space where information could be identified and retrieved. Shortly after the turn of the millennium the Web 2.0 became more interactive and enabled increased potentials to nurture connections and engage in communities. People began to move more and more of their social activities to digital platforms. Thereby, the term connectivity originally indicating computer transmissions quickly assumed the denotation of users engaging in *new public squares* accumulating social capital and building relationships (Tapscott & Williams, 2006).

Digital connectivity is not a new phenomenon in the health‐care domain but has substantially increased during the COVID‐19 pandemic where interactions moved from joint territorial location of health professionals and their patients to digital platforms that are facilitating remote care through applications such as messaging services, videoconferencing interfaces, and joint access to electronic databases (Hollander & Carr, 2020). During the last two decades the sociology of health and illness has theorised this new medical cosmology under the notion of *e‐scaped medicine* (Nettleton, 2004). *Scapes* are conceived as fluid geographies where practices of patient follow‐up and information are no longer confined to territorial location (e.g. consultation rooms) but ubiquitously accessible through digital connectivity. Digital connectivity reconfigures traditional conceptions of copresence where doctors and patients had been co‐located and found themselves and accessible to their naked senses, experiencing visual body language, human voice, touch, and smell (Goffman, 1983). Digital connectivity links remote persons through tangible artefacts (e.g. apps or web interfaces) that project dispersed objects (e.g. blood results) and enable collaborative actions (e.g. clinical encounter) across spaciotemporal situations. The possibility to connect to medical services from any location, at times, is perceived as step towards decreasing patients’ dependency on the clinic but has also wider social implications as the following example illustrates.

The reconfiguration of relationships has been investigated in a recent study on platform encounters in HIV care (Marent et al., 2018, 2021). In this case, a health platform (accessible through a clinical web interface and smartphone app) was implemented to facilitate asynchronous communication and information exchange between clinicians and stable HIV patients. The new possibilities of digitised forms of connectivity reconfigured conceptions of closeness and privacy and created ambivalent feelings among clinicians and HIV patients. In some instances, distance‐presence was enacted as form of connectivity that facilitates support when needed and enables close and intimate relations. However, in other cases, particularly where patients were newly diagnosed or had more serious health problems, the spatiotemporal blurring of closeness was negated while preference for corporal presence and the need to sense human proximity was expressed. Other studies have also highlighted how users of online forums need to become *acclimatised* to new forms of absent‐presence in order to facilitate intimacy and relationships in *digital atmospheres* (Tucker & Goodings, 2017). Current applications of the concepts of affectivity and senses provide fruitful directions to understand how feelings of closeness/distance materialise within digital spaces (Lupton, 2017). Theorising connectivity also requires us to acknowledge possibilities for protecting against unwanted connections. In the case of the HIV health platform (Marent et al., 2018), these aspects were particularly discussed in relation to privacy. Digital networks can facilitate potential of *invisibility* that might protect the privacy of HIV patients by keeping them physically distant from clinics. In contrast, however, digital networks can also increase visibility and transparency of patients. Issues of privacy require new attention to the question of how connections can be inhibited, dissolved, or forgotten in digital assemblages (Esposito, 2017).

The studies highlighted above provide examples for the new and often invisible types of work that are involved in establishing new modes of connectivity between health‐care providers and service users. These require new competences to provide or access care through digital networks (e.g. communicating medical results). Furthermore, new sociomaterial practices reconfigure roles and responsibilities of professionals and patients and bring forth new understandings of what is considered as *good care*. In relation to privacy, digital forms of connectivity may increasingly render the patients invisible in the material space but, potentially, increase patients’ visibility through the data traces left while navigating the virtual space.

Under the focus *reconfiguration of relationships*, a sociomaterial approach towards digital health practices addresses the connections that evolve through digital networks. It is important to challenge solutionist assumptions that consider digital technologies as simple tools that make collaborations between health‐care providers and interactions between clinicians and patients more efficient. Rather, a sociomaterial approach enables to acknowledge the distinct qualities of different interaction environments (e.g. face‐to‐face, phone, videoconferencing, asynchronous messaging) and their affordances. Thereby, it can offer some guidance to orchestrate digital connectivity and to consider when (e.g. for which patient in what type of circumstances) and how (e.g. communication policies) different forms of digitised patient‐provider interactions may be integrated in care pathways. A broader interest in digital ecosystems concerns the question of how digital connectivity provides new forms of surveillance that require solid benefit‐risk analysis and joint efforts to secure privacy and trust of users.

### Instantaneity: Reconfigurations of control

Our third suggested focus for a sociomaterial approach to digital health involves asking the question: How are digital materialities entangled in the temporality of practice and reconfigurations of control? If we find ourselves still in the computer history museum, we could approach one of the microchips developed by Intel co‐founder Gordon Moore back in the 1960s. These microchips contain millions of transistors that are made from silicon. The materiality of the silicon allows electric currents to pass and magnify energy. This enables microchips to gain power for advanced computing and automation processes. As Gordon Moore predicted in his *law of acceleration* microchips will become increasingly efficient, doubling their computational power each year (Plesner & Husted, 2020). Throughout the past decades this law proofed accurate—but currently the physical limits of putting more transistors on microchips seems to be reached—and led to social theoretical attempts to account for accelerated temporalities (Wajcman, 2019) and unprecedented forms of instantaneous time (Urry, 2000). In combination with advanced algorithms computing processors enable task execution with high speed, often experienced as instantaneity. This new velocity is being increasingly exploited to delegate tasks between human and nonhuman actors, leading to distributed and ambiguous forms of control.

New temporalities and the reconfiguration of control have been subject of investigation in the sociology of heath and illness, particularly in relation to practices of self‐tracking and the self‐management of chronic disease (e.g. Marent et al., 2018; Pols et al., 2019). In entanglements formed by patients and digital technologies control over accomplishing certain health tasks is distributed. Thereby, temporalities of health practices are often reconfigured as attentive responses to prompts from the digital device. This has been outlined by Pols et al. (2019) who argued that *reflexive agency* in self‐tracking is exercised through continuous notifications from devices, which interrupt unreflexive, routinised or intuitive ways of acting. Mobile health devices, Miller (2021) argues, provide a *perpetual opportunism* where health can become a topic at any time in everyday life through automated feedback and instant prompts. This raises the question of whether digital patients or citizens are more active or more passive participants in care since lines of actions often do not presume intentionality. A sociomaterial approach can explore the new forms of control that emerge through human‐machine configurations.

Such an approach was undertaken by Schwennesen (2019) in the field of physical rehabilitation, where algorithmic systems take on the role of physiotherapists. In her ethnographic study, Schwennesen (2019) observed how patients after hip transplantation surgery were equipped with an algorithmic apparatus, consisting of a smartphone and app as well as five wireless sensors, which could be worn during home training. This apparatus could monitor patients’ progress in their physiotherapy, observe how they deviate from the optimal bodily movements during exercise and suggest corrections and adjustments in real‐time. This feedback appeared as instant text messages (e.g. ‘keep up the good work’) and visual images (e.g. exercise rated between one and three stars) on the smartphone screen as well as through digital voice corrections (e.g. ‘lift your knees to a higher position’).

Schwennesen’s sociomaterial approach, challenges perspectives where algorithms are understood as capable of *acting alone*. While the algorithmic system was designed to take on professional tasks in clinical practice (predictive diagnosis and treatment regimes in particular), Schwennesen charts the ways in which this *algorithmic authority* is, in fact, negotiated and sometimes broken down in use, arguing that agency does not derive from an essential quality of the algorithm itself but is produced through associations made between social and material *actants* (Latour, 2005) including algorithmic imaginaries, policies, sensors, smartphones, IT workers, private companies, municipalities, physiotherapists and patients. The algorithmic system needs to be adjusted and creatively *repaired* to build and maintain meaningful control operations that enable a productive (mutually constitutive) relationship between system and bodies undergoing rehabilitation. Schwennesen’s (2019) study demonstrates the need for a new mode of accountability focussing on how algorithmic systems come to work in medical practice. This differs both from a *transparency approach* (disclosure of factors that influence algorithmic decision‐making) and an approach based on identifying bias (embedded norms and values that may have discriminatory effects). Algorithmic systems are based on generative rules rather than clear regulatory rules that the entangled patient would clearly know and could intentionally comply with. Both the machine and the patient learn and adapt in the flow of agency, where calibration and the accomplishment of good care often require bypasses and the identification of loopholes.

Focussing on *reconfigurations of control* investigates the instantaneous forms of data flow and calculation, which create unprecedented control and regulating mechanisms. Algorithms embed new forms of control that can threaten to displace human judgement, decision‐making and even care. Applying this thinking to digital health research and innovation, specifically, requires attention to the new forms of agency in order to exploit their opportunities and prevent their potential negative consequences. The concept of *bounded automation* (Fleming, 2019) is useful to underline that in most processes of automation, control is negotiated and distributed between humans and non‐humans.

## STRATEGIES FOR ENGAGING IN DIGITAL HEALTH INNOVATION

We have demonstrated how a sociomaterial approach towards digital health pays attention to the combined and interrelated challenges of quantification, connectivity, and instantaneity to explore and negotiate the type of care we want to achieve through digital transformation. From these three foci, we now want to turn to the question of how sociomaterial researchers might engage directly in digital health innovation and transformation processes. In order to do this, we will elaborate more the onto‐epistemological foundations of sociomateriality by discussing the notions of ontological politics, relationality, and performativity and bring these into dialogue with key concepts from the digital innovation literature: participation, affordances and generativity (Nambisan et al., 2019). First, in considering ontologies as heterogenous, we argue that participation becomes an important strategy to account for multiple views and interests in the shaping and evaluation of digital heath. Second, we argue that the relational ontology corresponds with an affordance perspective that traces intertwinements of digital health along functions, users and embedding environments in order to assess the accomplishment of good care. Third, we argue that the performativity of practice requires research and innovation efforts to account for generativity and engage with the contingent, complex, and ongoing journeys through which digital health comes into being. These three strategies for engaging in digital health research and innovation work together constitutively. For example, participatory approaches reveal technological affordances and offer consideration of future possibilities, leading to generativity in the technology’s innovation pathway. Rather than engaging with one or two of the strategies outlined in the following, digital health research and innovation should embrace all three together, match their perspectives and negotiate their results in order to achieve meaningful innovations and take responsibility for novel forms in which care becomes configured.

### Participation

As we have argued, a sociomaterial approach conceives ontologies as heterogeneous and contingent. This brings them into the political arena, where alternative *worldings* can become articulated (Haraway, 2016, p. 76). Sociomateriality scrutinises the *ontological politics* through which realties come into existence, considering ontology, epistemology and ethics as deeply intertwined (Mol, 2013, p. 381). This requires taking‐up responsibility for the generation of knowledge and normative preferences enacted. Barad (2007) proposes an *ethico‐onto‐epistem‐ology* where we as researchers are part of particular materialisations that exist. Therefore, we are responsible for the articulation and configuration of the phenomena we study and need to recognise and include the knowledge, thinking and imagination of heterogeneous actors.

Participation, defined here as a deliberative and collaborative process for developing digital health interventions and underlying public policies, is regarded as central ethos in technology studies (Lezaun et al., 2017; Nielsen & Langstrup, 2018). Participatory practices are considered as strategic engines for innovation and the transparency and accountability of digital health innovation. Yet, participatory governance is a highly complex endeavour that is often too narrowly captured through conventual models and methods. This work, as Chilvers and Kearnes (2020, p. 349) point out, ‘relies on highly specific pregiven meanings, forms, and qualities of participation’ and, often, lacks engagement with the relational coproduction of sociomaterial ontologies. Therefore, the authors provide a novel framework for the ‘remaking of participation’ in technology innovation processes that attends to the emergent process and its uncertain outcomes. Their framework aims to *ecologize participation* by attending to the interrelation between diverse public engagements in societies and, thereby, account for multiple publics and broader processes through which knowledge and power are configured within spaces of participation. Participation as strategy recognises the need to engage with multiple collectives to continually review imaginaries, aims, and consequences of digital health. Rather than seeking to resolve contradictions and conflicts, this strategy embraces ambivalences and accounts for the inherent uncertainties of participatory processes. Such processes generate new possibilities of understanding and action. Therefore, participation provides a strategy through which research and innovation can take account of the ontological politics and agential cuts involved in digital health endeavours.

### Affordances

Sociomateriality’s relational ontology takes a critical stance towards the technological‐centric focus of research and policy, which itself reflects the nomenclature of commercial vendors who characterise digital health devices through bundles of features and product classes. As Faraj and Azad (2012) have pointed out, the superimposition of pre‐defined categories over technology‐in‐use leads to black boxing of key aspects of materiality and how these come to matter over time. Therefore, outcome‐based evaluations and related policy recommendations on the utilisation of digital health often face difficulties in elaborating the link between certain qualities of technologies and the clinical, economic, or user‐experience outcomes (Greenhalgh & Swinglehurst, 2011). This creates problems in guiding policy recommendations towards diffusion and scale‐up of technologies and requires *sociotechnical evaluation* approaches, as stressed by Marc Berg in the late 1990s (Berg, 1999). Therefore, scholars in science and technology studies and organisation science have widely adopted the notion of affordances (Parchoma, 2014). The concept of affordances enables the potentials and possibilities of technologies to be approached as contingent and practical accomplishments. It is the relationship between a certain technological aspect, the specific user and use contexts that enables or restrict the possibilities of agency. As Faraj and Azad (2012, p. 238) argue, affordances ‘are rooted in a relational ontology which gives equal play to the material as well as the social’. In this way, the concept of affordances does not refer to specific qualities or features of certain digital health devices nor their users. ‘An affordance is neither an objective property nor a subjective property’ as Gibson (1979, p. 129) elaborated. Rather, affordance allows us to understand the relationship between the environment (materiality) and its observer. Thus, affordances can be understood as the multifaceted relational structure between technological features (e.g. app with messaging service), embedding environments (e.g. chronic care pathways), and their participants (e.g. stable HIV patient) that enable or constrain practices of care. The concept of affordances highlights that the materiality of the technological device is distinct in relation to the ways in which it is embedded and used by different patients or their health‐care providers. Marent et al. (2021) adopt this approach to illustrate complex considerations when patients are enabled access to their personal medical data. For example, if a newly diagnosed HIV patient receives a blood test result on his app and graphs are illustrating how important medical parameters drop significantly, this could lead to feelings of uncertainty and helplessness. Particularly, if the patient does not receive an accompanying and reassuring personal message from his doctor, or opportunities to request phone or face‐to‐face consultations.

An affordance perspective allows investigating distinct materialisations of technologies and how these lead to different outcomes in different use contexts. Thereby, it can contribute towards an analysis of the wider societal implications of digital technologies in terms of empowerment, inclusion, and equity.

### Generativity

From a sociomaterial approach, we have elaborated that the boundaries of digital health are not pregiven or fixed but enacted in practices. The concept of *generativity* is increasingly adopted in technology and innovation studies to account for the contingent, complex, and ongoing journeys through which digital technologies come into being (Thomas & Tee, 2021). Digital technologies are generative through their *emergent design* that facilitates openness, distributedness, editability, recombinability, and transferability to a large, varied, and uncoordinated audience (Nambisan et al., 2019). From a sociological approach, Karin Knorr Cetina (1997) has conceptualised digital technologies as *epistemic things*. While epistemic things have material instantiations—with specific features and functionalities—they are question generating and continuously acquire new properties. Knorr Cetina stresses that epistemic things lack *object‐ivity* and *completeness of being*. They are best understood as continually unfolding structures that combine presence and absence. Through practices of use, epistemic things become adapted, and their functionalities are being changed. In Barad’s (2007, p. 177) terms, we can observe how, in intra‐actions with epistemic things, ‘new possibilities open up as others that might have been possible are now excluded: possibilities are reconfigured and reconfiguring.’

The generativity of sociomaterial practices requires us to approach digital health as a continuous process that begins before the technology itself is present and continues well into implementation and use phases. Once in use, platforms, apps, and other technological artefacts are in a continuous process of transformation. They require constant fixes, updates, and versions, not only because of technological change but also because of necessary sociocultural developments that accompany them. This requires ongoing engagement with actual practices where technology has to be tamed and adjusted (Schwennesen, 2019) to fit specific situations of usage. Digital technologies come to matter alongside sociocultural developments where users ascribe different functionalities across different fields of practice. Engaging with practices of design and use provides valuable insights into how distinct *actants* (Latour, 2005) approach, imagine, anticipate, and intra‐act in materialisations of digital health. Rather than looking backwards upon settled technological infrastructures, recognising generativity is part of a strategy that investigates concrete materialisations of digital health and stimulates acts of imaginations about desirable futures.

## CONCLUSION

In further elaborating a sociomaterial approach to digital health, this article provides a comprehensive understanding of how digitalisation affords practices of health and medicine to cope with and utilise the combined and interrelated challenges of increases in quantification, varieties of connectivity, and unprecedented modes of instantaneous calculation. We have argued that different forms of knowledge, relationships and control are produced through particular manifestations of digital health and, thereby suggested important foci through which to explore and negotiate the types of care we want to achieve through digital transformation. Situated within an agential realist tradition, we have also reflected upon the role we as sociologists play within the configuration of digital health futures. Drawing on the work of Barad in particular, we have argued that responsible agential cuts towards the materialisation of digital health require participatory efforts as well as the recognition of the affordances and the generativity of technology developments. These strategies can lay the foundations to reorient and sensitise innovation processes in order to create new possibilities and value‐centric approaches for promoting health in digital societies as opposed to promoting digital health per se.

## AUTHOR CONTRIBUTIONS

**Benjamin Marent**: Conceptualization (lead); Writing‐ original draft (lead); Writing‐review & editing (lead). **Flis Henwood**: Conceptualization (supporting); Writing‐original draft (supporting); Writing‐ review & editing (equal).

## Data Availability

Data sharing is not applicable to this article as no new data were created or analysed in this study.

## References

[shil13538-bib-0001] Armstrong, D. (2019). The social life of data points: Antecedents of digital technologies. Social Studies of Science, 49(1), 102–117. 10.1177/0306312718821726 30636567 PMC6902902

[shil13538-bib-0002] Barad, K. (2003). Posthumanist performativity: Toward an understanding of how matter comes to matter. Signs: Journal of Women in Culture and Society, 28(3), 801–831. 10.1086/345321

[shil13538-bib-0003] Barad, K. (2007). Meeting the universe halfway: Quantum physics and the entanglement of matter and meaning. Duke University Press.

[shil13538-bib-0004] Bennett, J. (2010). Vibrant matter: A political ecology of things. Duke University Press.

[shil13538-bib-0005] Berg, M. (1999). Patient care information systems and health care work: A sociotechnical approach. International Journal of Medical Informatics, 55(2), 87–101. 10.1016/s1386-5056(99)00011-8 10530825

[shil13538-bib-0006] Cecez‐Kecmanovic, D. , Galliers, R. D. , Henfridsson, O. , Newell, S. , & Vidgen, R. (2014). The sociomateriality of information systems: Current status, future directions. MIS Quarterly, 38(3), 809–830. 10.25300/misq/2014/38:3.3

[shil13538-bib-0007] Chilvers, J. , & Kearnes, M. (2020). Remaking participation in science and democracy. Science, Technology & Human Values, 45(3), 347–380. 10.1177/0162243919850885

[shil13538-bib-0008] Esposito, E. (2017). Artificial communication? The production of contingency by algorithms. Zeitschrift für Soziologie, 46(4), 249–265. 10.1515/zfsoz-2017-1014

[shil13538-bib-0009] Faraj, S. , & Azad, B. (2012). The materiality of technology: An affordance perspective. In P. M. Leonardi , B. A. Nardi , & J. Kallinikos (Eds.), Materiality and organizing: Social interaction in a technological world (pp. 237–258). Oxford University Press.

[shil13538-bib-0010] Faulkner, A. (2008). Medical technology into healthcare and society: A sociology of devices, innovation and governance. Palgrave Macmillan.

[shil13538-bib-0011] Faulkner, P. , & Runde, J. (2019). Theorizing the digital object. MIS Quarterly, 43(4), 1279–1302.

[shil13538-bib-0012] Finch, T. L. , Mort, M. , Mair, F. S. , & May, C. R. (2008). Future patients? Telehealthcare, roles and responsibilities. Health & Social Care in the Community, 16(1), 86–95. 10.1111/j.1365-2524.2007.00726.x 18181818

[shil13538-bib-0013] Fleming, P. (2019). Robots and organization studies: Why robots might not want to steal your job. Organization Studies, 40(1), 23–38. 10.1177/0170840618765568

[shil13538-bib-0014] Gibson, J. J. (1979). The ecological approach to visual perception of experimental psychology. Lawrence Erlbaum.

[shil13538-bib-0015] Goffman, E. (1983). The interaction order. American Sociological Review, 48(1), 1–17. 10.2307/2095141

[shil13538-bib-0016] Greenhalgh, T. , & Swinglehurst, D. (2011). Studying technology use as social practice: The untapped potential of ethnography. BMC Medicine, 9(1), 1–7. 10.1186/1741-7015-9-45 21521535 PMC3108909

[shil13538-bib-0017] Haraway, D. J. (2016). Staying with the trouble. Duke University Press.

[shil13538-bib-0018] Harman, G. (2016). Immaterialism: Objects and social theory. Polity Press.

[shil13538-bib-0019] Henwood, F. , & Marent, B. (2019). Understanding digital health: Productive tensions at the intersection of sociology of health and science and technology studies. Sociology of Health & Illness, 41(1), 1–15. 10.1111/1467-9566.12898 31599984

[shil13538-bib-0020] Hollander, J. E. , & Carr, B. G. (2020). Virtually perfect? Telemedicine for COVID‐19. New England Journal of Medicine, 382(18), 1679–1681. 10.1056/nejmp2003539 32160451

[shil13538-bib-0021] Hoyer . (forthcoming). Data‐intensive medicine. In A. Petersen (Ed.), Handbook of the sociology of health and medicine. Edward Elgar.

[shil13538-bib-0022] Jones, M. (2019). What we talk about when we talk about (big) data. The Journal of Strategic Information Systems, 28(1), 3–16. 10.1016/j.jsis.2018.10.005

[shil13538-bib-0023] Kitchin, R. (2014). The data revolution. Big data, open data, data infrastructures & their consequences. Sage.

[shil13538-bib-0024] Kivits, J. (2013). E‐health and renewed sociological approaches to health and illness. In K. Orton‐Johnson & N. Prior (Eds.), Digital sociology. Critical perspectives (pp. 213–226). Palgrave Macmillan.

[shil13538-bib-0025] Knorr Cetina, K. (1997). Sociality with objects: Social relations in postsocial knowledge societies. Theory, Culture & Society, 14(4), 1–30. 10.1177/026327697014004001

[shil13538-bib-0026] Latour, B. (2005). Reassembling the social: An introduction to actor‐network‐theory. Oxford University Press.

[shil13538-bib-0027] Law, J. (2009). Actor network theory and material semiotics. In B. S. Turner (Ed.), The new Blackwell companion to social theory (pp. 141–158). Blackwell.

[shil13538-bib-0028] Lemke, T. (2021). The government of things. New York University Press.

[shil13538-bib-0029] Lezaun, J. , Marres, N. , & Tironi, M. (2017). Experiments in participation. In U. Felt , R. Foche , C. A. Miller , & L. Smith‐Doerr (Eds.), The handbook of science and technology studies. MIT Press.

[shil13538-bib-0030] Lievevrouw, E. , Marelli, L. , & Van Hoyweghen, I. (2021). The FDA’s standard‐making process for medical digital health technologies: Co‐producing technological and organizational innovation. BioSocieties, 1–28. 10.1057/s41292-021-00232-w PMC8116827 34002115

[shil13538-bib-0031] Lupton, D. (2012). M‐health and health promotion: The digital cyborg and surveillance society. Social Theory & Health, 10(3), 229–244. 10.1057/sth.2012.6

[shil13538-bib-0032] Lupton, D. (2017). How does health feel? Towards research on the affective atmospheres of digital health. Digital Health, 3, 2055207617701276. 10.1177/2055207617701276 PMC6001181 29942587

[shil13538-bib-0033] Lupton, D. (2019). The thing‐power of the human‐app health assemblage: Thinking with vital materialism. Social Theory & Health, 17(2), 125–139. 10.1057/s41285-019-00096-y

[shil13538-bib-0034] Marent, B. , & Henwood, F. (2021). Digital health. In K. Chamberlain & A. Lyons (Eds.), Routledge international handbook of critical issues in health and illness (pp. 261–275). Routledge.

[shil13538-bib-0035] Marent, B. , & Henwood, F. , & EmERGE Consortium . (2021). Platform encounters: A study of digitised patient follow‐up in HIV care. Sociology of Health & Illness, 43, 1117–1135. 10.1111/1467-9566.13274 33818815

[shil13538-bib-0036] Marent, B. , Henwood, F. , & Darking, M. , & EmERGE Consortium . (2018). Ambivalence in digital health: Co‐designing an mHealth platform for HIV care. Social Science & Medicine, 215, 133–141. 10.1016/j.socscimed.2018.09.003 30232053

[shil13538-bib-0037] Miller, D. (2021). A theory of a theory of the smartphone. International Journal of Cultural Studies, 24(5), 860–876. 1367877921994574. 10.1177/1367877921994574

[shil13538-bib-0038] Mol, A. (2013). Mind your plate! The ontonorms of Dutch dieting. Social Studies of Science, 43(3), 379–396. 10.1177/0306312712456948

[shil13538-bib-0039] Monteiro, E. , & Parmiggiani, E. (2019). Synthetic knowing: The politics of the internet of things. MIS Quarterly, 43(1), 167–184. 10.25300/misq/2019/13799

[shil13538-bib-0040] Nambisan, S. , Wright, M. , & Feldman, M. (2019). The digital transformation of innovation and entrepreneurship: Progress, challenges and key themes. Research Policy, 48(8), 103773. 10.1016/j.respol.2019.03.018

[shil13538-bib-0041] Nettleton, S. (2004). The emergence of E‐scaped medicine? Sociology, 38(4), 661–679. 10.1177/0038038504045857

[shil13538-bib-0042] Nielsen, K. D. , & Langstrup, H. (2018). Tactics of material participation: How patients shape their engagement through e‐health. Social Studies of Science, 48(2), 259–282. 10.1177/0306312718769156 29676208 PMC5946664

[shil13538-bib-0043] Numerato, D. , Vochocová, L. , Štětka, V. , & Macková, A. (2019). The vaccination debate in the “post‐truth” era: Social media as sites of multi‐layered reflexivity. Sociology of Health & Illness, 41(S1), 82–97. 10.1111/1467-9566.12873 31599993

[shil13538-bib-0044] Orlikowski, W. J. (2007). Sociomaterial practices: Exploring technology at work. Organization Studies, 28(9), 1435–1448. 10.1177/0170840607081138

[shil13538-bib-0045] Østerlund, C. , Crowston, K. , & Jackson, C. (2020). Building an apparatus: Refractive, reflective, and diffractive readings of trace data. Journal of the Association for Information Systems, 21(1), 10–22. 10.17705/1jais.00590

[shil13538-bib-0046] Parchoma, G. (2014). The contested ontology of affordances: Implications for researching technological affordances for collaborative knowledge production. Computers in Human Behavior, 37, 360–368. 10.1016/j.chb.2012.05.028

[shil13538-bib-0047] Petersen, A. (2019). Digital health and technological promise: A sociological inquiry. Routledge.

[shil13538-bib-0048] Petersen, A. , Tanner, C. , & Munsie, M. (2019). Navigating the cartographies of trust: How patients and carers establish the credibility of online treatment claims. Sociology of Health & Illness, 41(S1), 50–64. 10.1111/1467-9566.12872 31599982

[shil13538-bib-0049] Petrakaki, D. , Hilberg, E. , & Waring, J. (2021). The cultivation of digital health citizenship. Social Science & Medicine, 270, 113675. 10.1016/j.socscimed.2021.113675 33434718

[shil13538-bib-0050] Pickering, A. (1995). The mangle of practice: Time, agency, and science. The University of Chicago Press.

[shil13538-bib-0051] Pickersgill, M. (2019). Digitising psychiatry? Sociotechnical expectations, performative nominalism and biomedical virtue in (digital) psychiatric praxis. Sociology of Health & Illness, 41(S1), 16–30. 10.1111/1467-9566.12811 30175439 PMC6849545

[shil13538-bib-0052] Piras, E. M. , & Miele, F. (2019). On digital intimacy: Redefining provider‐patient relationships in remote monitoring. Sociology of Health & Illness, 41(S1), 116–131. 10.1111/1467-9566.12947 31599992

[shil13538-bib-0053] Plesner, U. , & Husted, E. (2020). Digital organizing: Revisiting themes in organization studies. Red Globe Press.

[shil13538-bib-0054] Pols, J. , Willems, D. , & Aanestad, M. (2019). Making sense with numbers. Unravelling ethico‐psychological subjects in practices of self‐quantification. Sociology of Health & Illness, 41(S1), 98–115. 10.1111/1467-9566.12894 31599983 PMC6856866

[shil13538-bib-0055] Puig de La Bellacasa, M. (2017). Matters of care: Speculative ethics in more than human worlds. University of Minnesota Press.

[shil13538-bib-0056] Schultze, U. , van den Heuvel, G. , & Niemimaa, M. (2020). Enacting accountability in IS research after the sociomaterial turn(ing). Journal of the Association for Information Systems, 21(4), 10–835. 10.17705/1jais.00620

[shil13538-bib-0057] Schwennesen, N. (2019). Algorithmic assemblages of care: Imaginaries, epistemologies and repair work. Sociology of Health & Illness, 41(S1), 176–192. 10.1111/1467-9566.12900 31599986

[shil13538-bib-0058] Suchman, L. A. (2007). Human‐machine reconfigurations: Plans and situated actions (2nd ed.). Cambridge University Press.

[shil13538-bib-0059] Tapscott, D. , & Williams, A. D. (2006). Wikinomics: How mass collaboration changes everything. Penguin.

[shil13538-bib-0060] Thomas, L. D. , & Tee, R. (2021). Generativity: A systematic review and conceptual framework. International Journal of Management Reviews, 24(2), 255–278. 10.1111/ijmr.12277

[shil13538-bib-0061] Tucker, I. M. , & Goodings, L. (2017). Digital atmospheres: Affective practices of care in elefriends. Sociology of Health & Illness, 39(4), 629–642. 10.1111/1467-9566.12545 28326556

[shil13538-bib-0062] Urry, J. (2000). Sociology beyond societies: Mobilities for the twenty‐first century. Routledge.

[shil13538-bib-0063] van de Wiel, L. (2019). The datafication of reproduction: Time‐lapse embryo imaging and the commercialisation of IVF. Sociology of Health & Illness, 41(S1), 193–209. 10.1111/1467-9566.12881 31599989 PMC6856688

[shil13538-bib-0064] Wajcman, J. (2019). The digital architecture of time management. Science, Technology & Human Values, 44(2), 315–337. 10.1177/0162243918795041

